# Testicular seminomatous mixed germ cell tumor with choriocarcinoma and teratoma with secondary somatic malignancy: a case report

**DOI:** 10.1186/1752-1947-8-1

**Published:** 2014-01-01

**Authors:** Amandeep Aneja, Siddharth Bhattacharyya, Jack Mydlo, Susan Inniss

**Affiliations:** 1 Department of Pathology and Laboratory Medicine, Temple University Hospital, 3401 North Broad Street, Philadelphia, PA 19140, USA; 2 Department of Urology, Temple University Hospital, Philadelphia, PA 19140, USA

**Keywords:** Mixed germ cell tumors, Rhabdomyosarcoma, Sarcomatous component

## Abstract

**Introduction:**

Testicular tumors are a heterogeneous group of neoplasms exhibiting diverse histopathology and can be classified as seminomatous and non-seminomatous germ cell tumor types. Mixed germ cell tumors contain more than one germ cell component and various combinations have been reported. Here, we present a rare case of a mixed germ cell tumor composed of seminoma, choriocarcinoma and teratoma with a secondary somatic malignancy.

**Case presentation:**

A 31-year-old Caucasian man presented with splenic rupture to our hospital. A right-sided testicular swelling had been present for 6 months and his alpha-fetoprotein, beta-human chorionic gonadotropin, and lactose dehydrogenase were increased. An ultrasound of his scrotum revealed an enlarged right testis with heterogeneous echogenicity. Multiple hypervascular lesions were noted in his liver and spleen. He underwent transcatheter embolization therapy of his splenic artery followed by splenectomy and right-sided orchiectomy. A computed tomography scan also showed metastasis to both lungs. During his last follow up after four cycles of cisplatin-based chemotherapy, the level of tumor markers had decreased, decreases in the size of his liver and pulmonary lesions were noted but new sclerotic lesions were evident in his thoracolumbar region raising concern for bony metastasis.

**Conclusions:**

Prognosis of testicular tumor depends mainly on the clinical stage, but emergence of a sarcomatous component presents a challenge in the treatment of germ cell tumors and the histological subtype of this component can be used as a guide to specific chemotherapy in these patients.

## Introduction

Testicular tumors are a heterogeneous group of neoplasms exhibiting diverse histopathology, variable clinical course and prognosis [1]. Of these tumors, 30 to 50% are classified as mixed germ cell tumors (GCT) [2] and several studies have assessed the frequency of various histological elements seen in these tumors [3,4]. The combination of seminoma and choriocarcinoma is reported to be extremely rare. Further, the presence of a sarcomatous component (SC) in a GCT is an infrequent phenomenon with great implications on clinical outcome and prognosis [5]. We report a rare case of mixed GCT with combination of seminoma, choriocarcinoma and teratoma with a secondary somatic malignancy of rhabdomyosarcoma.

## Case presentation

A 31-year-old Caucasian man was referred to our hospital for splenic rupture with active arterial extravasation. During clinical evaluation, he mentioned right testicular swelling; the swelling had been enlarging for 6 months. Transcatheter embolization therapy of the inferior pole of his splenic artery was performed for what was clinically thought to be a hypervascular metastasis in the spleen. Multiple hypervascular lesions were also noted in his liver. An ultrasound of his scrotum revealed an enlarged right testis with heterogeneous echogenicity measuring 5×4×3cm. Serum tumor markers revealed a beta-human chorionic gonadotropin (hCG) of 3804mIU/mL (<5mIU/mL), lactose dehydrogenase (LDH) of 196U/L (100 to 190U/L) and an alpha-fetoprotein (AFP) of 47.7ng/mL (0.0 to 9.0ng/mL). He underwent right-sided orchiectomy and splenectomy. No lymph node dissection was performed.

On gross pathological examination, a 5.1×5×2.5cm heterogeneous testicular mass, exhibiting tan-white to yellow to hemorrhagic areas was evident. No gross involvement of his tunica albuginea or spermatic cord was noted.

**Figure 1:**
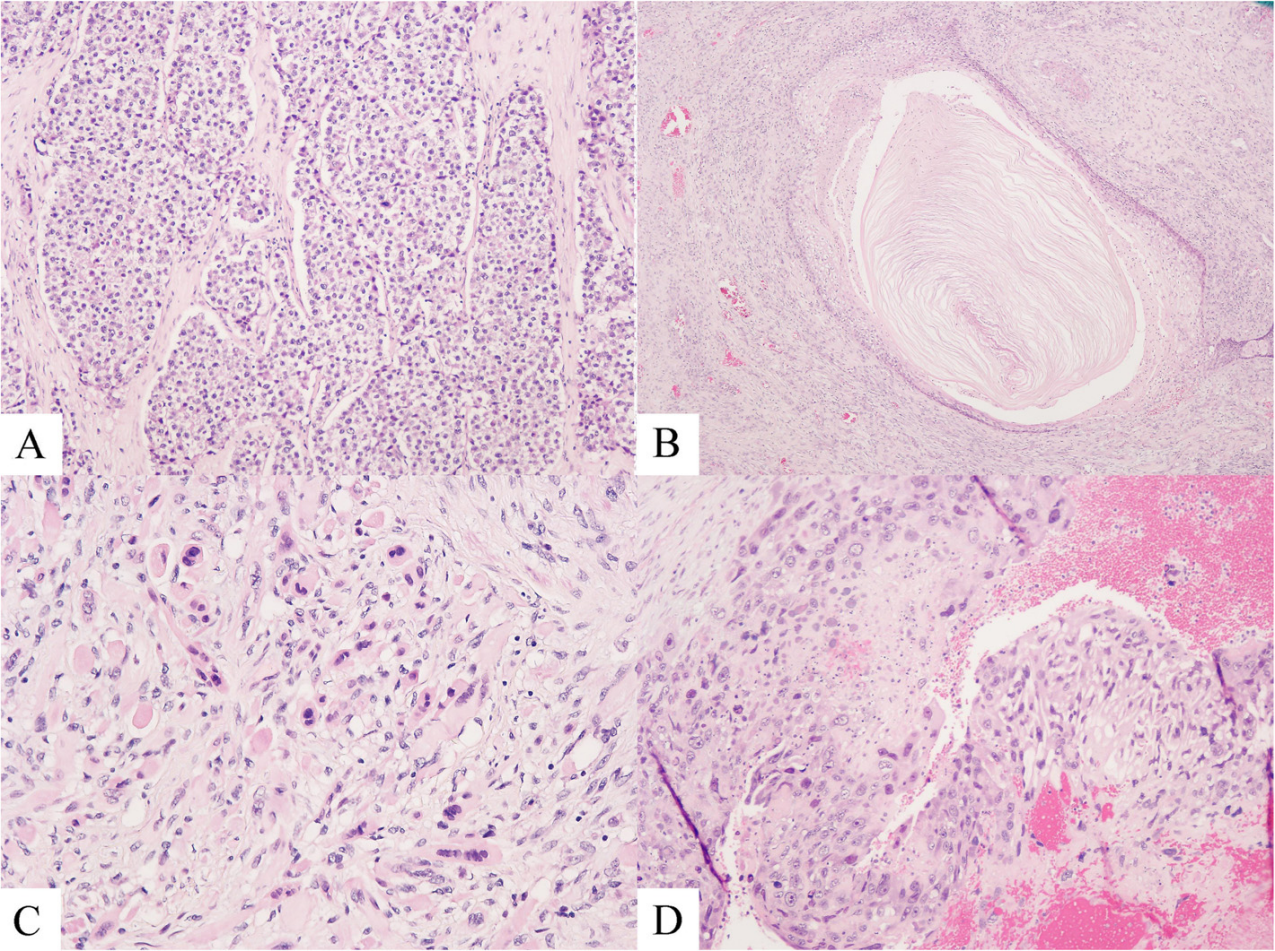
**Histological features of mixed germ cell tumor and sarcomatous component. (A)** Compact nests of tumor cells separated by fibrous septa, seminoma. Hematoxylin and eosin, 10×. **(B)** Keratinizing squamous epithelium, teratoma, hematoxylin and eosin, 4×. **(C)** Striated muscle cells with bizzare hyperchromatic nuclei, rhabdomyosarcoma. Hematoxylin and eosin, 20×. **(D)** Admixture of polygonal cells (cytotrophoblasts) and multinucleated cells (syncytiotrophoblasts) in a hemorrhagic background, choriocarcinoma. Hematoxylin and eosin, 10×.

**Figure 2:**
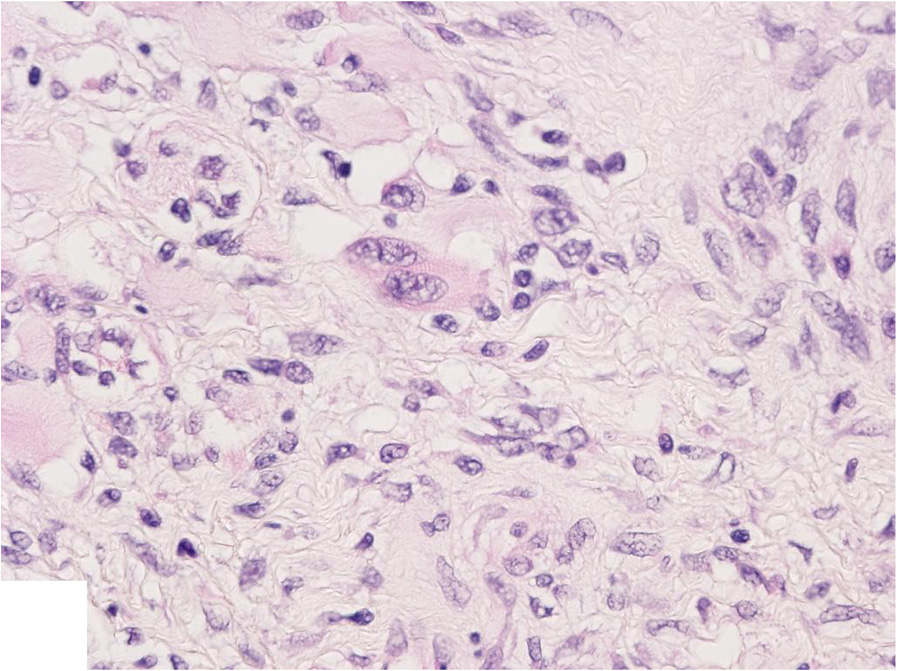
**Striated muscle cells, rhabdomyosarcoma.** Hematoxylin and eosin, 40×.

Histopathology showed a mixed GCT, confined to the testis containing seminoma, classic type (40%; Figure 1A), teratoma (40%; Figure 1B) with a secondary somatic malignancy (rhabdomyosarcoma; Figure 1C) and choriocarcinoma (20%; Figure 1D). The SC consisted of striated muscle cells with hyperchromatic bizarre nuclei and mitotic figures (Figure 2). Lymphovascular invasion and extension into the rete testis was observed. The epididymis was free of tumor. Intratubular germ cell neoplasia was also identified.

**Figure 3:**
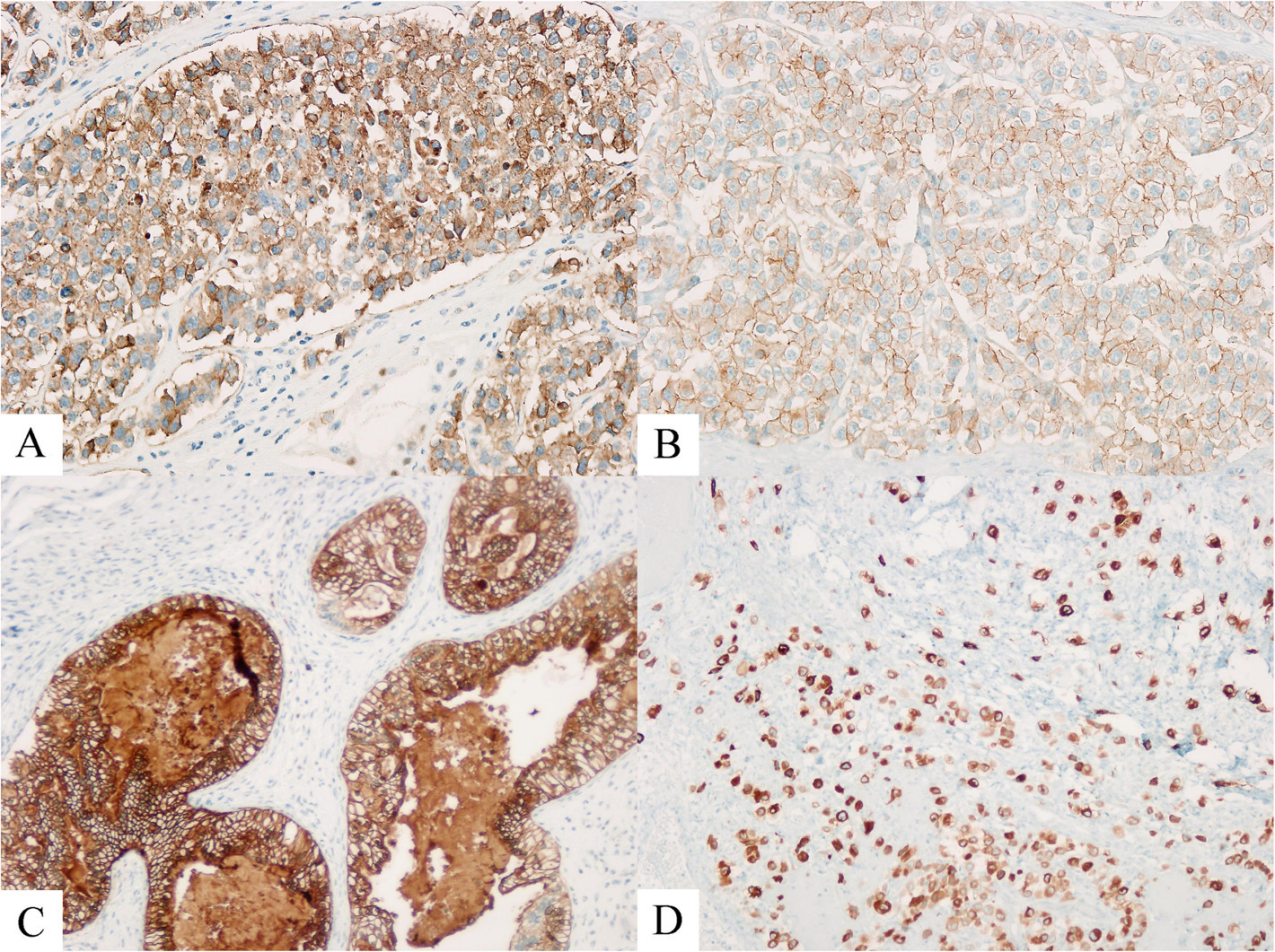
**Immunohistochemical stains. (A)** Seminoma, positive staining for placental alkaline phosphatase, 20×. **(B)** Seminoma, positive staining for c-Kit, 20×. **(C)** Teratoma, positive staining for epithelial membrane antigen, 10×. **(D)** Choriocarcinoma, positive staining for inhibin, 10×.

Immunohistochemistry stains displayed strong staining with placental alkaline phosphatase (Figure 3A) and c-kit (Figure 3B) in the seminomatous component. Epithelial membrane antigen (Figure 3C) and cytokeratin positivity were observed in the glandular component of the teratoma. The rhabdomyosarcoma showed positivity with vimentin and the choriocarcinoma was positive for inhibin (Figure 3D), cytokeratin and beta-hCG. Gross examination of his spleen showed four discrete foci suspicious for metastatic disease but microscopic examination revealed necrotic tissue without evidence of metastasis. Final pathological staging was pT2pNxpM0. However, computed tomography of his abdomen was suggestive of metastasis to his liver and both lungs making his diagnosis clinical stage IIIC.

He was placed on cisplatin-based chemotherapy and he has received four rounds to date. Bleomycin was omitted from the first round only because he was unable to perform pulmonary function tests; his exhalation time was too short and the end test criteria were not met. A repeat pulmonary function test 2 weeks later revealed normal air flow and normal diffusion capacity. By the end of the fourth cycle, his AFP had returned to normal and his beta-hCG had decreased to 137mIU/mL and LDH was 196U/L.

During his last follow up, we observed that his pulmonary and liver lesions had decreased in size but new sclerotic lesions had developed in his thoracolumbar spine which raised concerns of bony metastatic involvement. A biopsy of his L5 vertebral body however, showed no evidence of metastatic disease.

## Discussion

Several studies have reported specific combinations of different GCT elements in mixed GCT of testis [2,3]. Mostofi [3] in classifying more than 6000 testicular tumors found >1 histological patterns in approximately 60% of cases with the most frequent combination of embryonal carcinoma, yolk sac tumor and choriocarcinoma. Statistical analysis of possible combinations by Mosharafa *et al*. revealed 10 possible pair combinations with the strongest correlation between teratoma and yolk sac tumor [6]. To the best of our knowledge, the combination of seminoma, choriocarcinoma and teratoma with rhabdomyosarcoma as a secondary somatic malignancy has not been reported so far.

According to the World Health Organization, a teratoma with somatic malignancy can be defined as a teratoma containing a malignant component of a type typically encountered in other organs and tissues, for example sarcomas and carcinomas [7]. Absence of this combination of germ cell elements in separate studies by Malagón *et al*. and Guo *et al*. of 46 and 33 cases respectively of GCT with a SC further support this as a rare combination of germ cell elements exhibiting a SC [5,8].

There are several hypotheses regarding the origin of SC in GCT. As this component tends to occur in GCTs that contain a teratomatous component, it has been suggested that SC may arise from malignant transformation of teratomatous mesenchyme. However, reports of SC in GCT without teratomatous component point towards other mechanisms such as aberrant differentiation of primitive germ cells and de-differentiation of blastomatous stroma in yolk sac component [5,9].

The majority of the sarcomatous tumor of the testis is an element of germ cell neoplasia but, occasionally, can be seen as an invasion from a paratesticular sarcoma or as a primary intratesticular sarcoma. However, this is a rare occurrence. Stewart *et al*. found only six cases, of which four were paratesticular and two were testicular, in a 30-year review of intrascrotal rhabdomyosarcomas [10]. Erbay *et al*. while reporting a case of primary testicular rhabdomyosarcoma emphasized investigating for the teratomatous elements in the primary tumor due to the rarity of this entity [11]. They also advised serial serologic evaluation of beta-hCG and AFP to exclude undetected GCT metastases. However, it is also important to differentiate SC from focal atypical stromal proliferations commonly seen in teratoma [8]. These areas usually lack marked cytological atypia and do not form an expansile nodule whereas the sarcomatous cells show prominent cytologic atypia, brisk mitotic activity and complex growth pattern. The histological analysis in our case clearly showed latter areas present within the teratomatous component.

Development of SC presents a challenge in the treatment of testicular GCT. Malagón *et al*. reported a statistically significant difference in survival (P<0.001) for patients having a SC in their GCT when compared to age and stage-matched patients with GCTs without SC [5]. Many studies have revealed resistance to the cisplatin-based chemotherapy in GCTs with SC [12-15]. Donadio *et al*. emphasized in their study the importance of histological type of SC as a guide to specific chemotherapy in their patients, for example GCT with rhabdomyosarcoma responds well to a doxorubicin-based regimen whereas GCTs with secondary adenocarcinoma benefit from 5-fluorouracil-based chemotherapy [16]. Guo *et al*. suggested in their study that the SC confined to the primary testicular GCT may not have as high a risk of mortality as those at a comparable stage without a SC [8]. They further indicated that patients with SC in metastasis may benefit from sarcomatous-oriented drugs. Alternatively, SC may occur in primary testicular GCT or metastasis.

## Conclusions

We report the first known example of a GCT with an unusual combination of seminoma, choriocarcinoma and teratoma with a somatic malignancy. This case not only reflects the heterogeneity in the biology of GCT of the testis but also highlights the aggressiveness of these tumors and challenges in treatment due to the emergence of a SC in them.

## Consent

Written informed consent was obtained from the patient for publication of this manuscript and accompanying images. A copy of the written consent is available for review by the Editor-in-Chief of this journal.

## Abbreviations

AFP: Alpha-fetoprotein; GCT: Germ cell tumor; hCG: Human chorionic gonadotropin; LDH: Lactose dehydrogenase; SC: Sarcomatous component.

## Competing interests

The authors declare that they have no competing interests.

## Authors’ contributions

AA performed the gross examination of the specimen, AA and SB both conceived the case report, searched the literature and drafted the manuscript. SI performed the histopathological evaluation of the slides and made substantial revisions to the manuscript. JM operated on our patient and made revisions to the manuscript. All authors read and approved the final manuscript.
